# Derivation and validation of a machine learning-driven score to predict the diagnostic yield of endomyocardial biopsy

**DOI:** 10.1038/s41746-026-02421-y

**Published:** 2026-02-09

**Authors:** Christian Basile, Christian L. Polte, Piero Gentile, Entela Bollano, Araz Rawshani, Anders Oldfors, Charlotta Ljungman, Sven-Erik Bartfay, Pia Dahlberg, Clara Hjalmarsson, Marie Björkenstam, Elena Gualini, Antonio Cannatá, Patrizia Pedrotti, Andrea Garascia, Gianluigi Savarese, Aldo Pietro Maggioni, Kristjan Karason, Emanuele Bobbio

**Affiliations:** 1 Department of Clinical Science and Education, Södersjukhuset, Karolinska Institutet, Stockholm, Sweden; 2 Department of Advanced Biomedical Sciences, University of Naples “Federico II”, Naples, Italy; 3 ANMCO Research Center, Heart Care Foundation, Florence, Italy; 4 Departments of Clinical Physiology and Radiology, Sahlgrenska University Hospital, Gothenburg, Sweden; 5 Department of Molecular and Clinical Medicine, Institute of Medicine at Sahlgrenska Academy, University of Gothenburg, Gothenburg, Sweden; 6 De Gasperis Cardio Center, Niguarda Hospital, Milan, Italy; 7 School of Medicine and surgery, University of Milano-Bicocca, Milan, Italy; 8 Department of Cardiology, Sahlgrenska University Hospital, Gothenburg, Sweden; 9 Department of Clinical Pathology, Sahlgrenska University Hospital, Gothenburg, Sweden; 10 Institute of Biomedicine, The Sahlgrenska Academy at the University of Gothenburg, Gothenburg, Sweden; 11 Department of Cardiology, King’s College Hospital NHS Foundation Trust, Denmark Hill, London, UK; 12 Department of Cardiovascular Sciences, Faculty of Life Sciences & Medicine, King’s College - London, London, UK; 13 Transplant Institute, Sahlgrenska University Hospital, Gothenburg, Sweden

**Keywords:** Biomarkers, Cardiology, Diseases, Medical research

## Abstract

Despite its low diagnostic yield, endomyocardial biopsy (EMB) remains the gold standard for establishing a definitive diagnosis in many cardiomyopathies. We developed and validated a machine-learning–based score to predict the likelihood of diagnostic EMB using non-invasive data. We retrospectively analyzed 775 heart failure patients who underwent EMB. A random forest algorithm was selected for score development based on superior discriminative performance. The model was externally validated in an independent cohort (*n* = 171). The study population was predominantly male (72.1%), with half of the patients in NYHA class III–IV. EMB yielded a definitive diagnosis in 19.9% of cases, most commonly amyloidosis (50%). A predictive score (0-100 range) was derived from key non-invasive predictors. Right ventricular late gadolinium enhancement (LGE) on cardiac magnetic resonance emerged as the strongest predictor, followed by left ventricular and atrial LGE, NTproBNP levels, and renal function. The model demonstrated excellent discrimination, with an area under the curve of 0.92 (95% CI = 0.89–0.96) in cross-validation and 0.91 (95% CI = 0.86–0.98) in the testing set, with consistent performance on external validation (AUC 0.82, 95% CI = 0.76–0.89). This machine-learning-based score may provide a non-invasive tool to support EMB decision-making in clinical practice.

## Introduction

Heart failure (HF) is a heterogeneous clinical syndrome caused by a wide variety of underlying etiologies, and accurate etiology-based diagnosis is essential to guide appropriate therapeutic strategies^1^. Endomyocardial biopsy (EMB) is an invasive procedure developed in the 1960s for the early diagnosis and monitoring of heart transplant rejection^2^. Since then, this technique has become an important tool for the diagnosis and evaluation of various cardiac disorders, such as cardiomyopathies, myocarditis, and infiltrative diseases^3,4^.

In recent years, advanced imaging techniques such as cardiac magnetic resonance (CMR) have greatly enhanced the non-invasive diagnosis and prognostic assessment of various cardiac diseases^5^. Despite technical advancements in terms of tissue processing and analysis, the diagnostic yield of EMB remains low^6,7^. Predicting whether an EMB will provide a definitive diagnosis would be highly valuable, yet current evidence is insufficient to establish non-invasive factors as reliable predictors.

This study aimed to investigate how clinical and instrumental features relate to a diagnostic EMB outcome. Additionally, we sought to develop and validate a predictive scoring system using a machine learning (ML) approach.

## Results

### Baseline characteristics

A total of 775 HF patients undergoing diagnostic EMB as part of their diagnostic work-up were included in the derivation, i.e., Swedish cohort. The median age of the cohort was 52 (IQR 40-62) years, with 72.1% being male. Approximately half of the patients had experienced symptoms for more than three months, and 50% were classified as NYHA class III-IV (Table 1).

**Table 1:** Baseline characteristics of the enrolled population in the derivation cohort, stratified by endomyocardial biopsy result

	Overall population (*N* = 775)	Non-diagnostic (*n* = 621, 80.1%)	Diagnostic (*n* = 154, 19.9%)	SMD (%)
**Demographical**				
Index date year	2010 [2003, 2015]	2009 [2002, 2014]	2014 [2007, 2016]	8.1
Age^a^, years	52 [40,62]	49 [39,59]	60 [49,69]	59.6
Male sex^a^, %	72.1	71.8	73.4	3.5
History of drug abuse^a^, %	4.0	4.7	1.3	19.9
Symptoms duration^a^, %				37.9
<2 weeks	15.4	12.7	25.6	
2 weeks - 3 months	33.0	35.6	23.3	
>3 months	51.6	51.7	51.1	
**Clinical**				
Weight, kg	79.0 [69.0, 90.4]	80.0 [69.0, 93.0]	76.0 [67.0, 85.0]	26.1
Height, cm	176.0 [170.0, 182.0]	176.0 [170.0, 183.0]	174.0 [168.0, 180.0]	18.8
BSA, m^2^	1.95 [1.80, 2.12]	1.97 [1.80, 2.14]	1.91 [1.78, 2.03]	26.7
BMI^a^, kg/m^2^	25.3 [22.7, 28.9]	25.4 [22.7, 29.2]	24.9 [22.6, 27.7]	17.2
Obesity (BMI ≥ 30 kg/m^2^)	18.5	19.7	13.7	16.0
SBP^a^, mmHg	120 [106, 130]	120 [106, 135]	115 [106, 130]	11.7
DBP^a^, mmHg	75 [68,84]	77 [68,84]	70 [65,84]	21.6
Heart rate^a^, bpm	75 [65,90]	76 [66,90]	72 [61,84]	26.0
NYHA class^a^, %				9.4
I	14.2	14.5	13.0	
II	36.2	36.8	33.8	
III	42.0	41.2	45.5	
IV	7.5	7.4	7.8	
**Treatment (%)**				
ACEi/ARB/ARNi	42.1	43.1	37.8	10.8
Beta-blocker	48.7	49.6	44.5	10.2
MRA	21.9	21.9	21.8	0.2
Oral anticoagulant	19.9	20.6	16.8	9.8
Loop diuretics	33.4	33.4	33.6	0.5
Digoxin	7.8	9.2	1.7	33.5
Amiodarone	3.6	3.6	3.4	1.4
Steroids	6.8	7.3	5.0	9.2
Corticosteroid-sparing drugs	1.1	1.0	1.7	6.4
Pacemaker	8.5	6.8	15.6	28.3
ICD/CRT	11.9	10.6	16.9	18.2
Mechanic circulatory support				30.9
Not needed	97.7	98.4	94.8	
VAD	1.8	1.2	3.9	
ECMO	0.3	0.0	1.3	
IABP	0.3	0.3	0.0	
**Comorbidities (%)**				
Hypertension^a^	20.6	20.9	19.5	3.6
Diabetes^a^	7.1	6.8	8.4	6.3
Systemic disease^a^	9.3	8.2	13.6	17.5
Active infection^a^	8.3	8.9	5.8	11.6
Previous myocarditis^a^	0.9	1.0	0.6	3.5
Ischemic heart disease^a^	4.1	3.9	5.2	6.4
**Laboratory values**				
Plasma Sodium, mmol/L^a^	139 [137, 141]	140 [138, 141]	139 [136, 142]	15.3
Plasma Potassium, mmol/L^a^	4.3 [4.1, 4.6]	4.3 [4.1, 4.6]	4.3 [4.1, 4.6]	3.3
eGFR CKD-EPI^a^, mL/min/1.73m^2^	57.9 [46.8, 70.2]	59.7 [48.9, 70.9]	51.1 [37.3, 60.7]	35.9
Severe CKD (eGFR <30 mL/min/1.73m^2^)	6.1	4.5	12.4	28.6
C reactive protein^a^, mg/dL	7.0 [2.5, 18.0]	6.0 [2.5, 17.7]	7.0 [2.5, 19.0]	5.6
CK-MB, ng/mL	2.5 [1.5, 7.0]	2.3 [1.7, 6.0]	5.0 [1.0, 16.0]	1.5
TnT, ng/mL	31.9 [15.0, 77.7]	24.0 [14.0, 61.0]	56.0 [26.5, 208.0]	44.4
NTproBNP^b^, pg/mL	1790 [601, 4903]	1480 [465, 4002]	3235 [1522, 6857]	33.9
**ECG parameters**				
Atrial fibrillation/atrial flutter^b^, %	21.1	19.2	28.9	23.0
Other arrythmic presentations^b^, %				61.2
No	63.2	67.6	45.5	
I AVB	8.1	5.6	18.2	
II AVB	0.8	0.6	1.3	
III AVB	1.9	0.6	7.1	
VT/VF/Cardiac arrest	26.0	25.5	27.9	
QRS duration^b^, ms	104 [94, 122]	102 [94, 118]	108 [92, 134]	11.4
LBBB^b^, %	15.2	15.3	14.8	1.4
RBBB^b^, %	8.4	6.4	16.1	30.9
Q wave^b^, %	12.2	7.8	29.6	58.4
Low voltages^b^, %	8.5	3.4	29.2	74.7
T wave pseudoinversion^b^, %	5.5	0.0	27.9	88.0
ST segment change^b^, %	34.7	34.3	36.4	4.3
**Echo parameters**				
LVEDD, mm	59 [50,68]	62 [52,69]	49 [44,55]	93.4
LVEDV, mL	148 [106, 194]	159 [118, 206]	99 [74, 136]	67.9
LVESV, mL	90 [53, 143]	107 [66, 161]	50 [33,83]	83.3
LVEF (%)	39 [22,55]	35 [21,55]	45 [32,55]	41.2
Dilated LV, %	46.7	52.5	23.4	62.9
**CMR parameters**				
LVEDV, mL	203 [153, 290]	225 [161, 313]	175 [142, 229]	56.6
LVESV, mL	116 [76, 199]	132 [76, 234]	91 [73, 151]	57.9
LVEDVi^a^, mL/m^2^	128 [89, 182]	139 [97, 189]	104 [81, 151]	26.7
LVESVi^a^, mL/m^2^	86 [47, 122]	91 [53, 137]	62 [41,91]	42.0
LVEF^a^ (%)	41 [26,52]	38 [24,52]	43 [32,54]	35.9
LV stroke volume^a^, mL	76 [59,94]	76 [62,94]	75 [56,92]	4.4
RVEDV, mL	169 [136, 214]	177 [136, 215]	160 [139, 210]	4.8
RVESV, mL	94 [64, 136]	95 [64, 136]	96 [62, 138]	0.6
RVEDVi^a^, mL/m^2^	98 [76, 139]	98 [75, 132]	100 [76, 158]	18.7
RVESVi^a^, mL/m^2^	56 [39,88]	57 [38,88]	55 [41,91]	15.5
RVEF^a^ (%)	45 [34,53]	44 [34,53]	46 [34,52]	9.5
LV anterior segments – positive LGE, %	14.3	4.2	55.2	134.6
LV septal segments – positive LGE^a^, %	22.6	10.0	73.4	167.9
LV inferior segments – positive LGE^a^, %	19.1	6.4	70.1	173.4
LV lateral segments – positive LGE^a^, %	13.9	3.5	55.8	139.6
Insertion points - positive LGE^a^, %	3.9	4.8	1.4	19.8
RV - positive LGE^a^, %	18.1	4.3	61.6	153.8
Atria - positive LGE^a^, %	14.2	3.7	56.5	140.7
Pericardial disease, %	7.9	8.3	6.8	5.5
Hypertrophy^a^, %				102.4
No	79.7	90.0	48.0	
Regional	17.6	8.2	46.7	
Global	2.6	1.7	5.3	
**Angiography (%)**				
Angiography performed	59.5	61.4	51.6	19.8
Stenosis over 50%	7.3	7.3	7.0	1.1
**Right heart catheterization**				
RAp, mmHg	5.0 [2.0, 10.0]	4.0 [2.0, 9.0]	6.5 [3.0, 12.25]	22.7
RVSp, mmHg	32.0 [25.0, 41.0]	32.0 [25.0, 41.0]	34.0 [24.0, 41.0]	2.7
RVEDp, mmHg	6.0 [3.0, 10.0]	5.0 [2.5, 10.0]	7.0 [3.0, 11.0]	9.8
PASp, mmHg	32.0 [24.0, 42.0]	31.0 [24.0, 42.0]	33.0 [24.0, 42.5]	4.4
PADp, mmHg	14.0 [8.0, 20.0]	13.0 [8.0, 20.0]	15.0 [8.5, 19.0]	7.2
PAMp, mmHg	22.0 [15.0, 29.0]	21.0 [15.0, 29.0]	22.0 [16.0, 28.0]	6.3
PCWp, mmHg	12.5 [7.0, 20.0]	12.0 [7.0, 20.0]	14.0 [9.0, 20.0]	2.1
CO, L/min	4.4 [3.5, 5.6]	4.4 [3.5, 5.6]	4.3 [3.5, 5.5]	12.8
SVO_2_, %	66.0 [58.3, 71.5]	66.7 [58.0, 71.7]	63.1 [59.0, 70.9]	8.0

^a^variables included in machine learning models.

*ACEi* ACE inhibitor; *ARB* angiotensin-receptor blocker; *ARNi* angiotensin receptor-neprylisin inhibitor; *AVB* atrio-ventricular block; *BMI* body mass index; *BSA* body surface area; *CKD* chronic kidney disease; *CKD-EPI* Chronic Kidney Disease Epidemiology Collaboration; *CK-MB* creatine kinase MB; *CO* cardiac output; *CRT* cardiac resynchronization therapy; *DBP* diastolic blood pressure; *ECMO* extracorporeal membrane oxygenation; *EF* ejection fraction; *eGFR* estimated glomerular filtration rate; *GLS* global longitudinal strain; *IABP* intra-aortic balloon pump; *ICD* implantable cardioverter defibrillator; *LBBB* left bundle branch block; *LGE* late gadolinium enhancement; *LV* left ventricle; *LVEDD* left ventricle end-diastolic diameter; *LVEDV* left ventricle end-diastolic volume; *LVEDVi* left ventricle end-diastolic volume indexed; *LVEF* left ventricle ejection fraction; *LVESV* left ventricle end-systolic volume; *LVESVi* left ventricle end-systolic volume indexed; *MRA* mineralocorticoid receptor antagonist; *NYHA* New York Heart Association; *PADs* pulmonary artery diastolic pressure; *PAMp* pulmonary artery mean pressure; PASp pulmonary artery systolic pressure; *PCWp* pulmonary capillary wedge pressure; *RAp* right atrial pressure; *RBBB* right bundle branch block; *RV* right ventricle; *RVEDp* right ventricle end diastolic pressure; *RVEDV* right ventricle end-diastolic volume; *RVEDVi* right ventricle end-diastolic volume indexed; *RVEF* right ventricle ejection fraction; *RVESV* right ventricle end-systolic volume; *RVESVi* right ventricle end-systolic volume indexed; *RVSp* right ventricle systolic pressure; *SBP* systolic blood pressure; *SVO2* venous oxygen saturation; *TnT* troponin T; *VAD* ventricular assist device; *VF* ventricular fibrillation; *VT* ventricular tachycardia.

Among the 775 patients, EMB yielded a diagnostic result in 19.9% of cases, with amyloidosis accounting for 49% of the histological diagnoses (Table 2). Patients with a diagnostic EMB differed from those with a non-diagnostic EMB in most baseline characteristics. Those with diagnostic EMB were older, had a shorter duration of symptoms, lower body mass index (BMI), estimated glomerular filtration rate (eGFR), blood pressure, and heart rate, and were more frequently in need of mechanical circulatory support. They also had higher N-terminal pro–B-type natriuretic peptide (NTproBNP) levels, higher left ventricular ejection fraction (EF), and more frequent late gadolinium enhancement (LGE) lesions and regional hypertrophy on cardiac magnetic resonance (CMR) (Table 1).

**Table 2:** Histological representation for the diagnostic endomyocardial biopsies in the derivation cohort

Histological diagnosis (*n*, %)	
Amyloidosis	75, 49.0%
Granulomatosis inflammation	29, 18.7%
Giant cell myocarditis	24, 15.5%
Lymphocytic myocarditis	10, 6.3%
Adipose tissue infiltration	6, 4.0%
Anthracycline-induced damage	3, 2.0%
Glycogen storage disease	2, 1.3%
Mitochondrial disease	2, 1.3%
Danon syndrome	1, 0.7%
Right heart lymphoma	1, 0.7%
Iron overload cardiomyopathy	1, 0.7%

The external validation, i.e., Italian cohort consisted of 171 patients who had similar demographic characteristics, with a median age of 47 (IQR 33–62) years and 67.8% male patients. However, a higher proportion of diagnostic EMB results were observed (38.0%), with amyloidosis comprising 30.7% of these diagnoses. A detailed summary of the baseline characteristics of the external validation cohort is provided in Supplementary Table S1.

### Selection of the best-performing machine learning algorithm

**Fig. 1:**
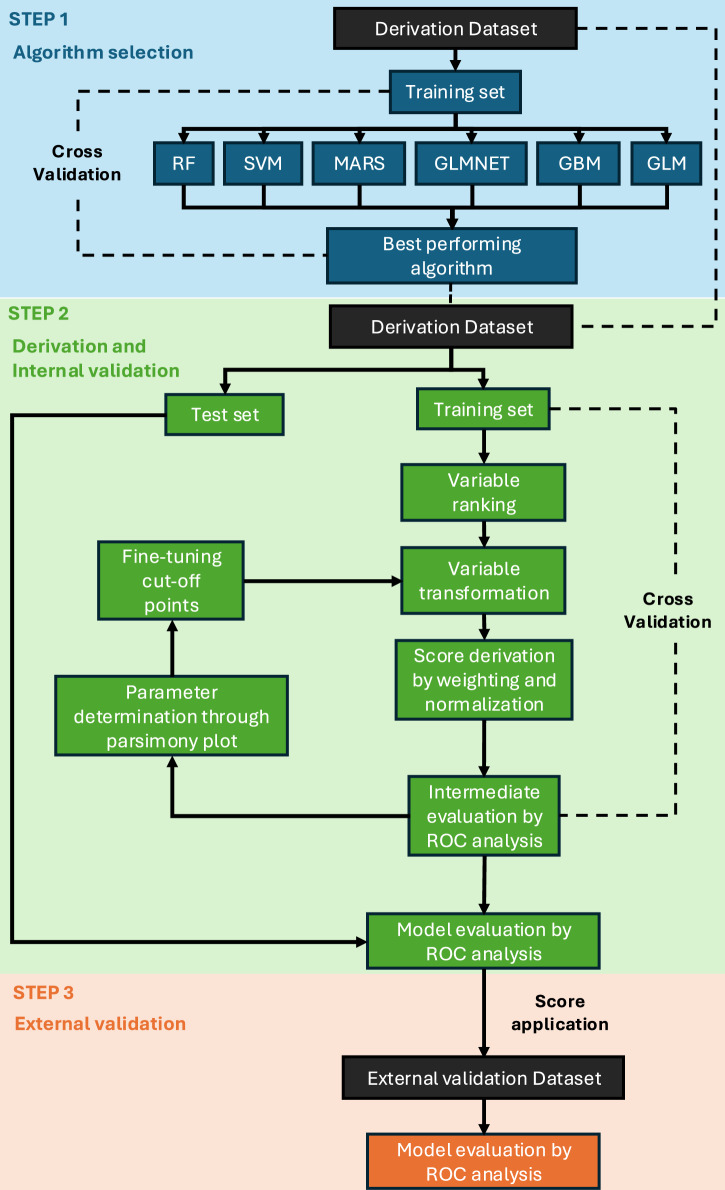
Score derivation and validation framework. GBM, gradient boosting machines; GLM, general logistic regression; GLMNET, generalized linear models via penalized maximum likelihood; MARS, multiple adaptive regression spline; RF, random forest; ROC, receiver operating characteristic; SVM, support vector machines.

In the first step, Random Forest (RF), Support Vector Machine (SVM), Multivariate Adaptive Regression Splines (MARS), Generalized Linear Model with Elastic Net Regularization (GLMNET), Gradient Boosting Machine (GBM), Generalized Linear Model (GLM) were evaluated using 10-fold cross-validation on the derivation dataset (Fig. 1), with the variables marked in Table 1 being included in the ML models. Among all tested algorithms, RF demonstrated the highest performance, achieving a median Area Under the Curve (AUC) of 0.95 (IQR 0.94–0.97), along with superior accuracy, Cohen’s Kappa coefficient, and sensitivity (Table 3).

**Table 3:** Comparison of the evaluated machine learning algorithms in the derivation cohort

Algorithm	Specificity	Sensitivity	AUC	Accuracy	Kappa
**RF**	0.64 [0.60–0.72]	1.00 [1.00–1.00]	0.95 [0.94–0.97]	0.93 [0.92–0.94]	0.76 [0.72–0.78]
**MARS**	0.71 [0.67–0.74]	0.98 [0.97–0.99]	0.91 [0.90–0.94]	0.91 [0.90–0.92]	0.70 [0.68–0.72]
**SVM**	0.69 [0.67–0.75]	0.98 [0.97–0.99]	0.95 [0.93–0.96]	0.92 [0.91–0.93]	0.74 [0.72–0.76]
**GBM**	0.69 [0.67–0.73]	0.98 [0.97–1.00]	0.94 [0.93–0.95]	0.92 [0.91–0.93]	0.73 [0.72–0.77]
**GLM**	0.74 [0.73–0.79]	0.96 [0.94–0.98]	0.90 [0.87–0.94]	0.90 [0.89–0.91]	0.69 [0.66–0.71]
**GLMNET**	0.66 [0.61–0.73]	1.00 [0.97–1.00]	0.94 [0.93–0.96]	0.92 [0.91–0.93]	0.73 [0.69–0.75]

*AUC* area under the curve; *GBM* gradient boosting machines; *GLM* general logistic regression; *GLMNET* generalized linear models via penalized maximum likelihood; *MARS* multiple adaptive regression spline; *RF* random forest; *ROC* receiver operating characteristic; *SVM* support vector machines.

Being the best-performing algorithm, RF was selected for the second step of the derivation process (Fig. 1).

**Fig. 2:**
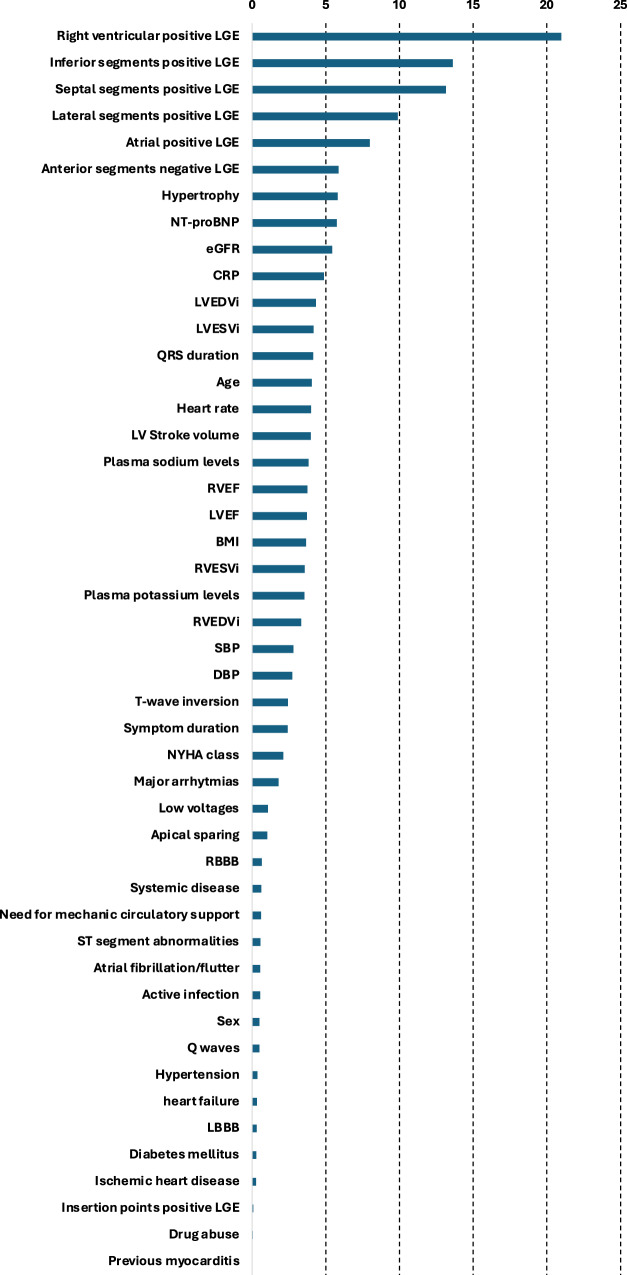
Feature importance of the random forest algorithm. BMI, body mass index; CRP, C-reactive protein; DBP, diastolic blood pressure; eGFR, estimated glomerular filtration rate; LBBB, left bundle branch block; LGE, late gadolinium enhancement; LVEDVi, left ventricular end-diastolic volume indexed; LVEF, left ventricle ejection fraction; LVESVi, left ventricular end-systolic volume indexed; NYHA, New York Heart Association; RBBB, right bundle branch block; RVEDVi, right ventricle end-diastolic volume indexed; RVEF, right ventricle ejection fraction; RVESVi, right ventricle end-systolic volume indexed; SBP, systolic blood pressure.

Fig. 2 presents the relative importance of different factors in predicting a diagnostic EMB of the RF algorithm in the training set. Overall, the top five factors to predict a diagnostic EMB in descending order of importance were the presence of a CMR LGE lesion in the right ventricle, a CMR LGE lesion in the left ventricular inferior, septal, and lateral segments, and a CMR LGE lesion in the atria. These five CMR features had the greatest importance on the diagnostic outcome compared with the remaining predictors.

### Score derivation and internal validation

**Fig. 3:**
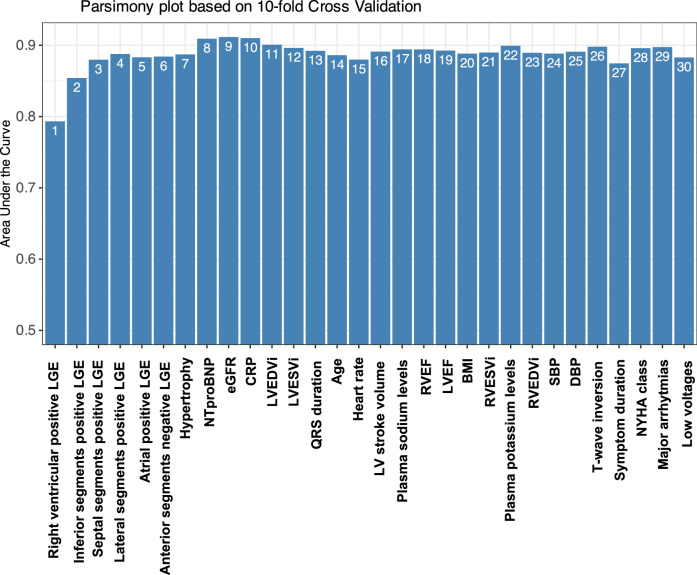
Parsimony plot of the selected variables. BMI, body mass index; CRP, C-reactive protein; DBP, diastolic blood pressure; eGFR, estimated glomerular filtration rate; LBBB, left bundle branch block; LGE, late gadolinium enhancement; LVEDVi, left ventricular end-diastolic volume indexed; LVEF, left ventricle ejection fraction; LVESVi, left ventricular end-systolic volume indexed; NYHA, New York Heart Association; RBBB, right bundle branch block; RVEDVi, right ventricle end-diastolic volume indexed; RVEF, right ventricle ejection fraction; RVESVi, right ventricle end-systolic volume indexed; SBP, systolic blood pressure.

To identify the minimum set of variables for model development, candidate predictors were ranked by importance, and a parsimony plot was generated (Fig. 3). The highest AUC was achieved with the top nine variables, including CMR-detected LGE lesions and myocardial hypertrophy, NT-proBNP levels, and eGFR. These variables were ultimately incorporated into the model. Following model fine-tuning, the final score was developed, ranging from 0 to 100 points (Table 4). Among the predictors, positive LGE in the right ventricle emerged as the strongest contributor to the score, adding 19 points, followed by positive LGE in the inferior, lateral, and septal segments of the left ventricle, no LGE in the anterior segments of the left ventricle, myocardial hypertrophy, elevated NT-proBNP, and reduced eGFR.

**Table 4:** Score for the selected feature in the Random Forest algorithm from the derivation cohort

Variables	Data driven		Fine tuned	
	Values	Points	Values	Points
**RV positive LGE**	No	0	No	0
	Yes	17	Yes	19
**Inferior segments positive LGE**	No	0	No	0
	Yes	12	Yes	12
**Septal segments positive LGE**	No	0	No	0
	Yes	10	Yes	10
**Lateral segments positive LGE**	No	0	No	0
	Yes	8	Yes	10
**Atrial positive LGE**	No	0	No	0
	Yes	4	Yes	3
**Anterior segments positive LGE**	No	10	No	10
	Yes	0	Yes	0
**Hypertrophy at CMR**	No	0	No	0
	Regional	8	Regional	9
	Global	19	Global	17
**eGFR**	<48.9	0	<30	0
	48.9-57.7	1	30-60	2
	57.7-68.5	2		
	≥68.5	5	≥60	4
**NT-proBNP levels**	<1450	0	<1500	0
	1450-3040	8	1500-3000	5
	3040-5290	10	3000-5000	7
	≥5290	15	≥5000	15

*CMR* cardiac magnetic resonance; *eGFR* estimated glomerular filtration rate; *LGE* late gadolinium enhancement; *NT-proBNP* N-terminal prohormone of brain natriuretic peptide; *RV* right ventricle.

Internal validation using 10-fold cross-validation on the training set (AUC 0.92, 95% CI 0.89–0.96) and the testing set (AUC 0.91, 95% CI 0.89–0.96) showed a robust and consistent (Venkatraman’s test *p*-value = 0.86) predictive performance (Supplementary Fig. S1).

**Fig. 4:**
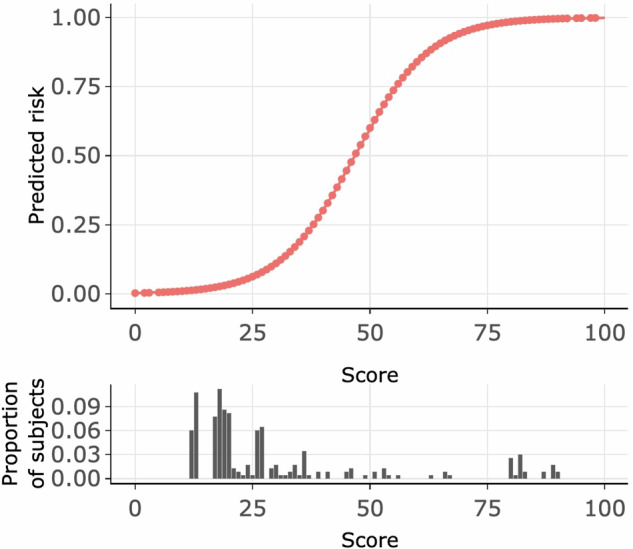
Distribution of the derived risk score and associated predicted risk. Proportion of patients with a specific risk score and predicted risk compared with points attainable in the final score.

Table 5 and Fig. 4 summarize the probability of obtaining a diagnostic EMB across various score thresholds in the testing set, with a cut-off at 60 points achieving the highest positive predictive value (100%) (Table 5).

**Table 5:** Score cut-off with their respective parameters in the testing set

Score cut-off (≥)	Predicted Risk (≥)	Percentage of patients (%)	Accuracy (95% CI)	Sensitivity (95% CI)	Specificity (95% CI)	PPV (95% CI)	NPV (95% CI)
0	1.3%	100	19.8% (19.8–19.8%)	100% (100–100%)	0% (0–0%)	19.8% (19.8–19.8%)	100% (100–100%)
10	1.3%	100	19.8% (19.8–19.8%)	100% (100–100%)	0% (0–0%)	19.8% (19.8–19.8%)	100% (100–100%)
20	3.5%	56	60.8% (54.7–66.8%)	91.3% (82.6–97.8%)	53.2% (46.2–60.2%)	32.6% (28.9–36.6%)	96.2% (92.3–99.1%)
30	11.1%	29	85.8% (81–90.1%)	87% (76.1–95.7%)	85.5% (80.1–90.3%)	60% (51.3–69.2%)	96.4% (93.5–98.8%)
40	32.9%	19	92.7% (89.2–95.7%)	78.3% (65.2–89.1%)	96.2% (93.5–98.9%)	84.1% (73.5–94.4%)	94.7% (91.9–97.3%)
50	63%	15	91.8% (88.8–94.8%)	67.4% (54.3–80.4%)	97.8% (95.7–99.5%)	88.9% (78.6–97.3%)	92.4% (89.4–95.3%)
60	88.4%	12	92.2% (89.2–94.8%)	60.9% (45.7–73.9%)	100% (100–100%)	100% (100–100%)	91.2% (88.2–93.9%)
70	98.4%	10	90.5% (87.9–93.5%)	52.2% (39.1–67.4%)	100% (100–100%)	100% (100–100%)	89.4% (86.9–92.5%)
80	98.4%	10	90.5% (87.5–93.1%)	52.2% (37–65.2%)	100% (100–100%)	100% (100–100%)	89.4% (86.5–92.1%)
90	99.5%	2	81% (80.2–82.3%)	4.3% (0–10.9%)	100% (100–100%)	100% (100–100%)	80.9% (80.2–81.9%)
100	100%	1	80.2% (80.2–80.2%)	0% (0–0%)	100% (100–100%)	100% (100–100%)	80.2% (80.2–80.2%)

*NPV* negative predictive value; *PPV* positive predictive value.

### External validation

**Fig. 5:**
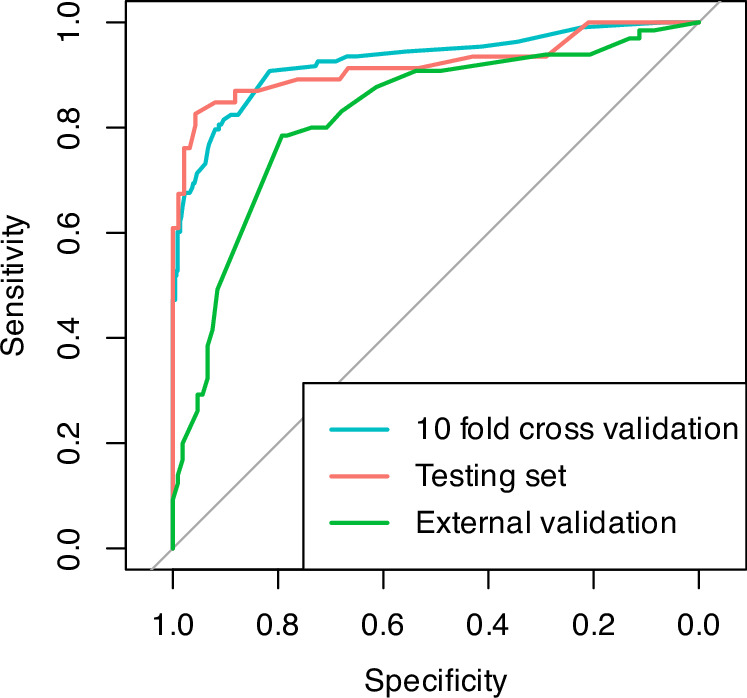
Discriminative performance of the derived risk score across different datasets. ROC curves for the derived score in the training set using 10-fold cross-validation, in the testing set and in the external validation cohort.

The external validation of the score using an independent cohort from a different institution achieved an AUC of 0.82 (95% CI 0.76–0.89) (Fig. 5). This performance, while slightly lower than the AUC observed in the testing set (0.91), demonstrated reasonable discriminatory power. The difference in AUCs between the testing and external validation sets was not statistically significant, as indicated by Venkatraman’s test (*p*-value = 0.06). Using the predefined cutoff of 60 points, the score achieved a comparable positive predictive value (Supplementary Table S2).

In terms of clinical utility, Decision Curve Analysis (DCA) showed that the score provided a positive Net Benefit compared with a ‘treat-all’ or ‘treat-none’ strategy across a broad range of threshold probabilities (22% to 82%) (Supplementary Fig. S2, left panel).

For the misclassification analysis, a detailed characterization of prediction errors is provided in Supplementary Table S3. The model returned a false negative result in 46 patients. The vast majority (82.6%) of these missed cases were non-amyloidosis etiologies. Phenotypically, these patients presented with ‘intermediate’ scores (median 51, IQR 36–64) and lacked specific high-risk features found in correctly identified cases, such as atrial and right ventricular LGE. False positive results were rare (*n* = 5). Paradoxically, these patients exhibited very high LGE burden (100% prevalence of LGE in the right ventricle, inferior, and lateral segments), which drove the score above the threshold despite a non-diagnostic biopsy.

Given the relatively low prevalence of diagnostic biopsies, we also assessed performance using Precision-Recall curves. The score showed good discrimination with a precision–recall AUC (PR-AUC) of 0.83 in the internal validation test set and 0.72 in the external validation cohort. A detailed analysis of performance metrics across scores 30–70 is provided in Supplementary Table S4. Statistically, the optimal balance between sensitivity and precision (highest F1-score and Balanced Accuracy) was observed at a score of 40–50. However, the pre-specified cut-off of 60 prioritized specificity ( > 95%) and Positive Predictive Value (PPV) ( > 79%), ensuring a high safety margin for identifying candidates for invasive biopsy.

### Sensitivity analyses

**Fig. 6:**
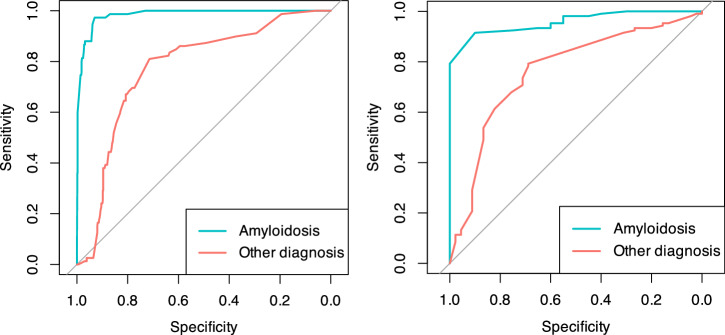
Diagnostic performance of the derived risk score for amyloidosis. ROC curves for the derived score in the overall dataset using 10-fold cross-validation for the prediction of an amyloidosis-positive endomyocardial biopsy vs other diagnosis (on the left) and in the external validation set (on the right).

In the sensitivity analysis on different EMB diagnoses using 10-fold cross-validation on the derivation dataset, the score significantly outperformed in predicting cardiac amyloidosis (AUC 0.98, 95% CI 0.97-0.99) compared with other EMB diagnoses (AUC 0.77, 95% CI 0.72-0.82; Venkatraman’s test for two unpaired ROC curves, *p* < 0.01). This better performance in predicting cardiac amyloidosis was confirmed in the external validation dataset (amyloidosis AUC 0.96, 95% CI 0.93–0.99; other EMB diagnoses AUC 0.76, 95% CI 0.68-0.85; Venkatraman’s test, *p* < 0.01) (Fig. 6).

When quartiles of enrollment were considered, no significant difference was present for AUC when stratified by quartile of enrollment neither in the derivation nor the external validation dataset (Supplementary Table S5), the same was accurate when considering patients with a shorter vs longer symptom duration (symptom duration ≤ 3 months AUC 0.90, 95% CI 0.85-0.94; symptom duration >3 months AUC 0.94, 95% CI 0.89-0.98; Venkatraman’s test, *p* = 0.08).

When the score model was compared with the complete random forest model in the internal validation test, the complete RF model achieved an AUC of 0.95, slightly superior to that of the point score (AUC 0.91) (Supplementary Fig. S3). While the RF model demonstrated superior calibration (Brier score 0.051 vs 0.081) and higher precision in the top 20% risk decile (PPV 87.2% vs 72.2%), the precision-recall curves showed extensive overlap, indicating that the majority of the diagnostic signal was captured in both models. In the external cohort, the complete RF model achieved an AUC of 0.83, comparable to that of the point score (AUC 0.82) (Supplementary Fig. S4). Furthermore, the Brier scores and PPVs in the top 20% risk decile were similar between the point score and complete RF models (0.184 vs 0.181 for the Brier Score, and 77.1% vs 82.9% for PPV).

In the sensitivity analysis excluding patients with amyloidosis, the DCA score retained a positive albeit less prominent Net Benefit across the probability threshold range of 22% to 56% (Supplementary Fig. S2, right panel).

Lastly, we assessed the influence of era and data missingness on our results. As detailed in Supplementary Fig. S5, the completeness of CMR and biomarker data improved significantly over the study period. Sensitivity analyses confirmed the score’s robustness despite these temporal shifts. In the “modern” cohort (2011–2018, *n* = 360), the score achieved an AUC of 0.90 (95% CI 0.86–0.94), which was statistically comparable to that of the historic cohort (AUC 0.89; *p* = 0.75). In the complete case sensitivity analysis, in which only patients with no missing data (*n* = 203) were considered, the score maintained a similar AUC of 0.93 (95% CI 0.87–0.98), with stable results despite imputation.

## Discussion

We derived, internally and externally validated in a different country, an ML-driven clinical score to predict the diagnostic yield of EMB in patients with HF (Graphical abstract). In this setting, we showed that: 1) EMB leads to a definitive diagnosis in only ~20% of cases involving HF of unknown etiology; 2) patients with a diagnostic EMB tend to have a more severe clinical phenotype; and 3) the primary predictors of a diagnostic EMB were specific LGE features on CMR, along with eGFR and NT-proBNP levels.

The score performed best in predicting cardiac amyloidosis, while still demonstrating reasonable discriminatory power in identifying other myocardial diseases.

In this study, we evaluated the diagnostic yield of EMB in a large cohort of patients presenting with HF of uncertain etiology. The diagnostic rate for EMB was only 19.9% in the Swedish cohort, which is consistent with prior studies reporting diagnostic yields ranging from 20% to 30% in non-transplant HF populations^8–11^. All our EMBs were obtained from the right ventricle. A recent systematic review of observational studies found that EMBs from both the right and left ventricles have similar diagnostic yields, with right ventricular biopsies showing a more favorable safety. Despite its unique value in identifying specific etiologies, many EMBs remain non-diagnostic, underscoring the need for better patient selection. In our cohort, patients with diagnostic EMBs had a more severe clinical profile, including older age, higher NT-proBNP levels, greater need for mechanical circulatory support, and slightly higher left ventricular EF. This aligns with previous studies indicating that EMB is particularly valuable in patients with rapidly progressive HF, where the likelihood of identifying inflammatory or infiltrative diseases is higher^12^, as well as in cases of HF with preserved EF, where EMB is more likely to yield a histological diagnosis compared with HF with reduced EF^13^.

The role of EMB in the etiological diagnostic work-up of HF has long been debated. While EMB remains the gold standard for diagnosing certain cardiac conditions, such as myocarditis and infiltrative cardiomyopathies, its clinical utility is limited by its invasive nature, associated risks (e.g., perforation, bleeding), and relatively low diagnostic yield. Several studies have attempted to refine the indications for EMB in HF, with the most widely accepted guidelines being those of the ESC and the American Heart Association (AHA), which recommend EMB only when it is likely to influence patient management^8^. Still, it should be noted that in specialized high-volume centers, the number of adverse events related to EMB is negligible ( < 1%)^14^.

Our study reinforces the value of EMB in specific HF phenotypes and clarifies how CMR can refine patient selection. The presence and pattern of LGE on CMR emerged as the strongest predictor of a diagnostic EMB, particularly when involving the right ventricle, the inferior, septal, and lateral LV segments, and the atria. This aligns with growing evidence supporting the use of LGE-CMR as a non-invasive marker of myocardial pathology. Several studies showed that LGE on CMR correlates with histopathological findings in cardiac sarcoidosis, myocarditis, and amyloidosis^15,16^. Our findings are even more relevant given that LGE is associated with an increased risk of all-cause mortality, HF hospitalization, and sudden cardiac death in patients with non-ischemic cardiomyopathy^17^. Interestingly, LGE in the anterior LV segments was inversely associated with a diagnostic EMB. Previous studies have reported weaker prognostic relevance of anterior LGE compared with other LV regions in dilated and hypertrophic cardiomyopathy^18,19^. Whether this is a spurious finding or underlines a different pathological route requires further research.

However, when considered alone, CMR still falls behind EMB in diagnostic accuracy^9,20,21^.

Our results suggest that CMR, in combination with other relevant clinical parameters, might be used as a screening tool to guide the decision on whether to proceed with an EMB. Given the invasive nature of EMB, its associated risks, and significant costs, improving patient selection through non-invasive imaging modalities could reduce the number of unnecessary, inconclusive biopsies and ensure that only high-yield cases are pursued. Additionally, CMR could guide EMB in imaging-guided biopsies, reducing sampling error by targeting specific LGE lesions^22^.

A major innovation of this study is the derivation, internal validation, and external validation of an ML-derived clinical score to estimate the probability that an EMB will be diagnostic. Using multiple algorithms, we identified nine clinical and imaging variables that were most predictive of a diagnostic EMB, including LGE in specific cardiac regions, NT-proBNP, and eGFR, with right ventricular LGE emerging as the strongest contributor.

The use of ML in cardiovascular diagnostics is an emerging field that has gained considerable attention in recent years. ML models have shown promise in predicting outcomes across a range of cardiovascular conditions, including coronary artery disease, arrhythmias, and HF^23–25^. By using the RF algorithm, we could develop a prediction model that achieved an AUC of 0.91 in the internal validation cohort, demonstrating excellent performance.

The score was particularly powerful for cardiac amyloidosis, where it achieved near-perfect discrimination (AUC up to 0.98). This is consistent with the “loud” phenotype of amyloidosis, characterized by diffuse and multi-chamber LGE, renal dysfunction, and markedly elevated NT-proBNP^26^. At the same time, modern practice increasingly allows non-biopsy diagnosis of transthyretin amyloidosis using bone scintigraphy and exclusion of monoclonal gammopathy. In this setting, the role of EMB is shifting toward patients with uncertain subtyping, suspected immunoglobulin light-chain amyloidosis (AL amyloidosis), or atypical presentations. The strongest predictors in the score (e.g., atrial and right ventricular LGE, NT-proBNP elevation) correspond to classic amyloid signatures, indicating that the model partially formalizes the established clinical gestalt. Importantly, however, the score provides value beyond confirming obvious cases: by offering a standardized, reproducible metric, it helps clinicians navigate ‘edge cases’ in which gestalt is equivocal. As shown in our DCA analyses, the score retains utility even in non-amyloid etiologies, acting as an objective gatekeeper to reduce low-yield biopsies and supporting clinical judgment when presentation is ambiguous (Text Box 1).

We acknowledge that the diagnostic landscape has evolved substantially with the adoption of bone scintigraphy for non-invasive identification of transthyretin cardiac amyloidosis (ATTR amyloidosis). Our score is not intended to replace these modalities. Instead, it provides support in the ‘residual’ population in whom non-invasive testing is negative or inconclusive. In such cases, particularly when AL amyloidosis, inflammatory cardiomyopathy, or atypical phenotypes remain possible, the decision to pursue EMB is challenging and often subjective. By quantifying the probability of obtaining a diagnostic specimen, the score helps clinicians determine when invasive risks are justified and when conservative management is more appropriate, thereby improving consistency in modern EMB decision-making.

Different score cut-offs yielded different performance profiles (Supplementary Table S4). Because the primary aim of the score is to identify patients in whom EMB is *most likely* to be diagnostic, we selected a cut-off of 60. While thresholds in the 40–50 range provided the highest statistical efficiency (F1-score ~0.73–0.81), we recommend a cut-off of 60 for clinical practice. In the context of an invasive procedure such as EMB, where avoiding futile risk is paramount, prioritizing PPV is clinically relevant. At the 60-point threshold, the score achieved high specificity ( > 95%) in both cohorts, functioning as a safe and pragmatic *rule-in* tool. This cut-off yielded the highest PPV and specificity while still maintaining good overall accuracy, Negative Predictive Value (NPV), and acceptable sensitivity.

Our etiology-specific sensitivity analysis (Fig. 6) confirmed that predictive performance is not uniform across all disease entities. Discrimination was highest for cardiac amyloidosis, likely reflecting its distinctive and “loud” phenotype, diffuse, multi-territory LGE, renal impairment, and markedly elevated NT-proBNP, all features heavily weighted in the algorithm. Conversely, performance for other diagnoses (primarily lymphocytic myocarditis and sarcoidosis) was more modest. These inflammatory conditions often present with subtler, patchy LGE or lower biomarker elevation, yielding intermediate scores (typically 30–50) that may not reach the 60-point rule-in threshold. This nuance is important in clinical practice: while a score ≥60 strongly predicts a diagnostic biopsy, lower or intermediate scores do not exclude inflammatory cardiomyopathies, and a lower biopsy threshold may be appropriate when clinical suspicion remains high.

A frequent criticism of simplified scores is that they sacrifice accuracy by ignoring non-linear interactions captured by more complex ML models. In our study, the full RF model outperformed the point score in internal validation, particularly in the highest-risk strata; however, this advantage was significantly lower in external validation, where both models performed similarly (AUC 0.82 vs. 0.83). This suggests that the score retains most of the relevant diagnostic signal while offering far greater usability. Beyond discrimination, the score provided meaningful clinical utility. In decision curve analysis, it conferred a clear Net Benefit across a wide probability range (22–82% overall; 22–56% in non-amyloid patients), supporting its role in guiding biopsy decisions, especially in equivocal cases.

A practical illustration highlights the potential impact: consider a 45-year-old man with HF with reduced EF (EF 35%), two months of symptoms, and non-specific LGE on CMR. A clinician might estimate his pre-test probability of a diagnostic biopsy at ~30%, below the usual threshold for pursuing an invasive procedure. However, the ML-Score assigns him 62 points, shifting him into a high-yield category. The biopsy confirms lymphocytic myocarditis, prompting immunosuppressive therapy and recovery of EF. Without the score, he might have been managed conservatively as “idiopathic DCM,” missing an opportunity for disease-specific treatment.

This study has, despite its strengths, several limitations. First, the broad time frame of patient enrollment does not consider the improvement in EMB preparation and evaluation over time, nor does it consider the improvement in imaging methods used, novel potential risk markers, or changed diagnostic work-up. Nonetheless, it’s important to note that the SMD for the index year between patients with diagnostic and non-diagnostic biopsies was less than 10% (8.1%), suggesting a minimal difference in the distribution of this variable. Furthermore, when results were stratified by quartile of enrollment, no significant difference emerged.

Second, as a single-center study, the results may not be widely applicable, being influenced by local EMB practices. However, the external validation performed in a different institution from a different nation showed consistent results.

Third, specific or more refined medical histories were not collected, although they may have contributed to prediction. Furthermore, many variables used to train the model in the internal validation cohort were not available in the external validation cohort, since the ethical permission was only requested for the variables needed to calculate the score in that cohort, and the analysis comparing the complete RF model vs the point score model was restricted to the variables already selected in the score model. Our derivation cohort spans an extended period (1994–2018), during which referral thresholds and CMR protocols evolved. We addressed this by employing imputation to handle early-era missingness and by performing multiple sensitivity analyses. While stable performance across these sensitivity analyses supports the validity of the score, and the external validation cohort had no missing data, we cannot entirely rule out score inflation due to the imputation method.

The strong predictive contribution of LGE location likely reflects two mechanisms: it identifies the presence and extent of myocardial pathology, and it increases the technical likelihood of sampling the affected tissue. Because current guidelines recommend directing EMB toward LGE-positive regions, this relationship partly reflects successful sampling. This should not be viewed as a limitation but rather as a key feature of the score, which aims to identify patients in whom the combination of disease burden and target accessibility makes EMB worthwhile. Conversely, disorders with patchy or subtle LGE patterns (e.g., myocarditis) yielded lower scores and lower diagnostic rates, reflecting their inherently higher risk of sampling error.

Finally, the number of patients with inflammatory cardiomyopathies, particularly lymphocytic myocarditis, was limited in our cohort. As a result, the model’s performance in this subgroup could not be robustly assessed. Furthermore, our misclassification analysis highlights specific ‘blind spots.’ While the score was highly sensitive for amyloidosis, it was less sensitive for inflammatory cardiomyopathies, which often present with subtler imaging features and intermediate scores. Clinicians could therefore consider a lower biopsy threshold in suspected inflammatory cases. Conversely, false positives were associated with extensive, diffuse LGE; in these cases, a high score with a negative biopsy may reflect sampling error or ‘burnt-out’ fibrotic disease where specific histological markers are no longer present. Further evaluation in larger, dedicated cohorts with suspected inflammatory etiologies is warranted to determine the generalizability of the score in this clinical context.

Nevertheless, we believe our study offers robust and valuable insights. To the best of our knowledge, this is the first study to use ML to assess the relative importance of various factors in predicting the diagnostic outcome of an EMB in patients with HF of unknown etiology.

In clinical practice, the overall diagnostic yield of an EMB was low. Using a user-friendly scoring system based on nine key factors, we accurately predicted the likelihood of a diagnostic result. Our findings highlight the importance of CMR in evaluating HF of uncertain etiology, especially when EMB is being considered.

Box 1 Clinical guidance for interpreting the score in different scenarios

**Table:** 

Clinical context		Amyloidosis-Suspected		Myocarditis-Suspected
**Clinical features**		Older age, HFpEF, LVH, multi-chamber LGE, elevated NT-proBNP		Younger age, acute/subacute onset, viral prodrome, chest pain, arrhythmia-predominant presentation
**Score**	≥60	EMB likely to be diagnostic		
	40-60	EMB considered if prior tests remain inconclusive	EMB recommended when suspicion remains high	
	<40	EMB unlikely to be diagnostic

## Methods

### Study design, patient population and ethical aspects

This retrospective cohort study included HF patients who underwent EMB due to myocardial disease of unknown etiology. The derivation cohort consisted of 775 consecutive patients identified from the Sahlgrenska University Hospital Heart Failure and Biopsy Registry (Gothenburg, Sweden) between September 1994 and December 2018. This registry consecutively enrolls all adult patients referred to the tertiary Heart Failure center for diagnostic EMB due to suspected cardiomyopathy or HF of unknown etiology. The external validation cohort included consecutive patients from Niguarda Cardiomyopathy Registry (Milan, Italy) between January 2007 and December 2024 who underwent EMB for suspected infiltrative or inflammatory cardiomyopathy and had complete data for all variables required to calculate the score.

The study conforms to the principles outlined in the Declaration of Helsinki and was approved by the Gothenburg County Regional Ethical Committee and Swedish Ethical Review Authority (Dnr 286-18 and 2023-02475-02). Informed consent was waived due to the retrospective nature of the study, as approved by the local ethics committees.

### Data source and definitions

Demographic data, symptoms, and EMB outcomes were collected, alongside results from laboratory tests, electrocardiography, echocardiography, angiography, right heart catheterization, and CMR imaging. Arrhythmia data were obtained through Holter electrocardiography and device interrogations. The baseline data were anchored to the index date, defined as the patient’s hospital admission for EMB. Detailed definitions in accordance with international guidelines were reported in Supplementary Table S6 and Supplementary Fig. S6^1,27–31^.

### Endomyocardial biopsy

Transvenous endomyocardial biopsy was performed in the supine position by cardiologists specialized in HF and transplantation using the standard Seldinger technique. Biopsies were obtained from the right ventricular septum using a disposable bioptome with fluoroscopic and echocardiographic guidance^32^. Five to seven specimens were obtained from each patient. Histopathological analysis included hematoxylin and eosin staining, and immunohistochemistry was implemented progressively during the study period, in accordance with evolving diagnostic standards.

### Outcome

The primary outcome was an EMB that provided a histopathological diagnosis.

### Machine learning model development

Since no known predictors of a diagnostic EMB exist, the score derivation and validation process was divided into three steps.

First, multiple machine learning algorithms were used to determine the best-performing algorithm, defined as the one leading to the highest median AUC through 10-fold cross-validation. The algorithms tested included RF, SVM, MARS, GLMNET, GBM, and GLM.

Second, the best-performing algorithm was used to derive an interpretable machine learning-driven clinical score^33^. The dataset was split into training (70%) and testing (30%) subsets. The training set was used to develop the score, incorporating 10-fold cross-validation for intermediate performance evaluation and hyperparameter tuning. The test set, serving as unseen data, was used to assess the final model performance.

To minimize overfitting, a parsimony approach was applied by plotting the top 30 features ranked by importance and selecting the smallest subset achieving the highest AUC. Feature thresholds were refined through a combination of data-driven methods and expert clinical judgment. A binary cutoff for the score was established to maximize positive predictive value, facilitating a “yes/no” classification for diagnostic utility.

Feature thresholds were initially generated using the AutoScore framework, which derives candidate cut-points from sample quantiles. To ensure clinical interpretability, these data-driven thresholds were subsequently refined through expert review to align with established pathological and guideline-based categories (e.g., CKD-EPI stages for eGFR, standardized NT-proBNP intervals, CMR-based definitions of hypertrophy, and AHA 17-segment LGE regional groupings).

### Statistical analysis

Continuous variables were reported as median and interquartile range (IQR), and categorical variables as frequencies (percentages).

Baseline characteristics of patients with a diagnostic vs. non-diagnostic EMB were compared by standardized mean difference (SMD), with a 10% SMD identifying an imbalance between the two groups’ baseline characteristics.

To assess whether simplifying the machine learning model to an additive score resulted in significant information loss, a head-to-head comparison was conducted. The final interpretable score was compared against the full RF model on the testing set using the same folds. Performance was evaluated using AUC, PR-AUC, calibration curves, and the Brier score. To test the robustness of this comparison, the analysis was repeated in the external validation cohort using a Random Forest model retrained on the specific variables included in the score.

To fully evaluate the score’s operating characteristics given the class imbalance (low event rate), we calculated the PR-AUC. Furthermore, we performed a threshold analysis calculating confusion matrices, Balanced Accuracy, and F1-scores across a range of score cut-offs (30 to 70) to identify different trade-offs between sensitivity and precision.

Later, the derived and internally validated score was externally validated using an independent cohort from a different country and a separate institution (Fig. 1).

To identify the clinical characteristics of patients for whom the model failed, a misclassification analysis was performed on the external validation cohort using the derived score cut-off of 60. Patients were stratified into true positives, false positives, true negatives, and false negatives, and their clinical and imaging features were compared to identify drivers of prediction errors.

Finally, to assess the clinical utility of the score beyond standard discrimination metrics, DCA was performed on the external validation cohort. ‘Net Benefit’ was assessed across a range of threshold probabilities to compare the predictive score against default strategies of performing a biopsy in all patients versus none.

Four sensitivity analyses were conducted: a first one involving the score for different EMB diagnoses, followed by a comparison of the corresponding AUC values, a second one assessing performance stability stratified by enrollment quartiles, a third one testing the score performance in patients with a symptom duration ≤ vs > 3 months, and a fourth one where a sensitivity DCA was also conducted, excluding patients with amyloidosis, to evaluate the utility in non-amyloid etiologies.

ROC curves were compared using Venkatraman’s test for two unpaired ROC curves.

To maximize statistical power and mitigate potential spectrum bias arising from historical data, missing values for clinical and imaging variables were handled using a RF-based single imputation approach (missRanger package in R). This method accounts for complex non-linear interactions between variables to impute missing data without reducing the sample size. To address the potential impact of evolving diagnostic protocols (e.g., lower availability of LGE in early years, or poorer image quality), two sensitivity analyses were performed: first, a modern era analysis where the cohort was stratified by enrollment year into ‘historic’ (1994-2010) and ‘modern’ (2011-2018) eras to assess performance stability in contemporary practice; second, a complete case analysis where the model was tested on the subset of patients with zero missing data to rule out bias introduced by imputation was performed. The external validation cohort had no missing data for the variables used to test the score.

All the statistical analyses were performed using R version 4.3.2. The level of significance was set to 5%, two-sided. The complete code used for preprocessing, model training, threshold derivation, and evaluation, as well as synthetic data, is available in the Supplementary materials.

## Supplementary information

Supplementary material

## Data Availability

The datasets generated and/or analysed during the current study are not publicly available due to ethical and privacy restrictions, but are available from the corresponding author on reasonable request.

## Code availability

The underlying code for this study is not publicly available but may be made available to qualified researchers upon reasonable request to the corresponding author.
