# Supplementary material for: Derivation and validation of a machine learning-driven score to predict the diagnostic yield of endomyocardial biopsy
Source: NPJ Digit Med. 2026 Feb 9;9:228. doi: 10.1038/s41746-026-02421-y (PMC12996545; doi:10.1038/s41746-026-02421-y)
Supplement: Supplementary file 1 — Supplementary material [file 41746_2026_2421_MOESM1_ESM.pdf]

Supplementary Table S1. Baseline characteristics of the enrolled population in the external validation cohort, stratified by endomyocardial biopsy result

	Overall population (N=171)	Non-diagnostic (n=106, 62.0%)	Diagnostic (n=65, 38.0%)	SMD (%)
**Demographical**				
Index date year	2017 [2014, 2020]	2017 [2013, 2020]	2018 [2014, 2020]	12.5
Age, years	47 [33, 62]	46 [34, 59]	50 [32, 65]	19.3
Male sex, %	67.8	70.8	63.1	16.4
**Laboratory values**				
eGFR CKD-EPI, mL/min/1.73m^2	87.0 [65.0, 112.0]	85.5 [62.7, 112.7]	87.0 [72.0, 111.0]	27.0
NTproBNP, pg/mL	2369 [920, 6115]	1209 [657, 2548]	5432 [3105, 10740]	52.7
**CMR parameters**				
LVEDV, mL	144 [113, 182]	149 [119, 194]	129 [108, 157]	40.1
LVESV, mL	74 [46, 103]	82 [48, 121]	68 [41, 89]	45.0
LVEDVi, mL/m^2	80 [62, 95]	83 [67, 102]	75 [59, 86]	40.2
LVESVi, mL/m^2	40 [26, 58]	44 [27, 67]	36 [25, 50]	40.0
LVEF (%)	46 [35, 61]	44 [30, 60]	49 [38, 63]	31.7
LV stroke volume, mL	66 [51, 84]	65 [52, 84]	66 [50, 84]	4.5
RVEDV, mL	124 [99, 152]	123 [99, 152]	127 [101, 150]	3.6
RVESV, mL	59 [41, 76]	59 [40, 74]	58 [43, 82]	1.1
RVEDVi, mL/m^2	67 [56, 82]	66 [57, 80]	67 [56, 84]	3.4
RVESVi, mL/m^2	32 [23, 40]	32 [23, 38]	32 [24, 44]	8.9
RVEF (%)	54 [43, 61]	55 [43, 61]	54 [42, 61]	6.3
LV anterior segments – positive LGE, %	55.0	52.8	58.5	11.4
LV septal segments – positive LGE, %	70.2	66.0	76.9	24.3
LV inferior segments – positive LGE, %	77.8	68.9	92.3	62.0
LV lateral segments – positive LGE, %	74.3	65.1	89.2	60.0
RV - LGE, %	50.3	36.8	72.3	76.3
Atria - LGE, %	14.6	7.5	26.2	51.3
Hypertrophy, %				48.7
No	77.2	84.9	64.6	
Regional	8.8	6.6	12.3	
Global	14.0	8.5	23.1	

CKD-EPI, Chronic Kidney Disease Epidemiology Collaboration; eGFR, estimated glomerular filtration rate; LGE, late gadolinium enhancement; LV, left ventricle; LVEDV, left ventricle end-diastolic volume; LVEDVi, left ventricle end-diastolic volume indexed; LVEF, left ventricle ejection fraction; LVESV, left ventricle end-systolic volume; LVESVi, left ventricle end-systolic volume indexed; RV, right ventricle; RVEDV, right ventricle end-diastolic volume; RVEDVi, right ventricle end-diastolic volume indexed; RVEF, right ventricle ejection fraction; RVESV, right ventricle end-systolic volume; RVESVi, right ventricle end-systolic volume indexed.

Supplementary Table S2. Performance of the derived score in the external validation cohort

Score cut-off (≥)	Percentage of patients (%)	Accuracy (95% CI)	Sensitivity (95% CI)	Specificity (95% CI)	PPV (95% CI)	NPV (95% CI)
60	14	70.2% (62.7-76.9%)	29.2% (18.6-41.8%)	95.3% (89.3-98.5%)	79.2% (57.8-92.9%)	68.7% (60.5-76.1%)

NPV, negative predictive value; PPV, positive predictive value

Supplementary Table S3. Misclassification analysis in the external validation cohort.

n	True Positive 19	False Positive 5	True Negative 101	False Negative 46	SMD (%)
Index date year	2017.00 [2012.00, 2022.00]	2015.00 [2013.00, 2019.00]	2017.00 [2013.00, 2020.00]	2018.00 [2015.00, 2020.00]	25.5
Age, years	66.00 [43.50, 75.00]	56.00 [54.00, 63.00]	45.00 [33.00, 58.00]	45.50 [31.00, 59.00]	61.9
Male sex, %	73.7	60.0	71.3	58.7	20
NYHA class, %					139.9
I	0.0	100.0	16.7	18.8	
II	40.0	0.0	50.0	43.8	
III	60.0	0.0	26.7	31.2	
IV	0.0	0.0	6.7	6.2	
eGFR CKD-EPI, mL/min/1.73m^2	83.00 [63.50, 96.00]	84.00 [75.00, 87.00]	86.00 [62.00, 113.00]	101.00 [74.75, 115.75]	23.9
NTproBNP, pg/mL	4580.00 [2985.00, 9700.00]	1168.00 [681.00, 2389.00]	1212.00 [654.00, 2560.00]	5668.50 [3127.25, 12700.00]	55.2
LVEDV, mL	128.00 [102.00, 153.00]	154.00 [138.00, 183.00]	148.00 [119.00, 194.00]	129.00 [116.75, 163.75]	30.4
LVESV, mL	68.00 [38.00, 85.50]	80.00 [79.00, 147.00]	84.00 [48.00, 119.00]	67.50 [43.25, 94.25]	40.1
LVEDVi, mL/m^2	62.00 [52.00, 82.50]	86.00 [86.00, 102.00]	83.00 [67.00, 102.00]	76.50 [62.00, 86.00]	47.9
LVESVi, mL/m^2	36.00 [22.00, 48.50]	50.00 [44.00, 82.00]	44.00 [27.00, 67.00]	36.00 [26.00, 49.50]	44.8
LVEF (%)	47.00 [42.00, 59.50]	42.00 [20.00, 49.00]	45.00 [31.00, 60.00]	49.50 [36.25, 63.75]	36.9
LV stroke volume, mL	66.00 [53.00, 77.00]	54.00 [45.00, 63.00]	65.00 [52.00, 84.00]	65.00 [50.00, 84.00]	32.4
RVEDV, mL	124.00 [86.00, 156.00]	108.00 [95.00, 123.00]	123.50 [101.25, 152.25]	127.00 [108.25, 147.00]	32.6
RVESV, mL	56.00 [34.00, 86.00]	41.00 [40.00, 87.00]	59.00 [40.75, 73.25]	58.50 [44.00, 76.00]	9.1
RVEDVi, mL/m^2	64.00 [48.50, 76.00]	59.00 [54.00, 69.00]	66.50 [57.00, 83.00]	68.50 [59.25, 84.75]	28.2
RVESVi, mL/m^2	32.00 [20.00, 45.00]	25.00 [20.00, 51.00]	32.00 [23.00, 38.00]	32.00 [24.25, 40.75]	8.6
RVEF (%)	54.00 [44.00, 60.00]	57.00 [29.00, 62.00]	55.00 [44.00, 60.00]	54.00 [42.50, 61.00]	20
LV anterior segments – positive LGE, %	36.8	20.0	54.5	67.4	58.3

LV septal segments – positive LGE, %	78.9	100.0	64.4	76.1	53.8
LV inferior segments – positive LGE, %	100.0	100.0	67.3	89.1	58.4
LV lateral segments – positive LGE, %	94.7	100.0	63.4	87.0	60.5
RV - positive LGE, %	94.7	100.0	33.7	63.0	108.5
Atria - positive LGE, %	47.4	20.0	6.9	17.4	51.4
Hypertrophy, %					87.3
No	26.3	80.0	85.1	80.4	
Regional	26.3	0.0	6.9	6.5	
Global	47.4	20.0	7.9	13.0	
EMB result (%)					156,3
Amyloidosis	63.2	0.0	0.0	17.4	
Non diagnostic	0.0	100.0	100.0	0.0	
Other	36.8	0.0	0.0	82.6	
Score	64.00 [61.00, 71.00]	61.00 [61.00, 64.00]	32.00 [27.00, 44.00]	51.00 [42.00, 52.00]	203,4

CKD-EPI, Chronic Kidney Disease Epidemiology Collaboration; eGFR, estimated glomerular filtration rate; LGE, late gadolinium enhancement; LV, left ventricle; LVEDV, left ventricle end-diastolic volume; LVEDVi, left ventricle end-diastolic volume indexed; LVEF, left ventricle ejection fraction; LVESV, left ventricle end-systolic volume; LVESVi, left ventricle end-systolic volume indexed; RV, right ventricle; RVEDV, right ventricle end-diastolic volume; RVEDVi, right ventricle end-diastolic volume indexed; RVEF, right ventricle ejection fraction; RVESV, right ventricle end-systolic volume; RVESVi, right ventricle end-systolic volume indexed.

Supplementary Table S4. Performance Metrics at Key Thresholds in Internal and External Validation Cohorts

Cohort	Threshold	TP	FP	TN	FN	Sensitivity	Specificity	PPV	NPV	Balanced Acc	F1 Score
**Internal validation**	**30**	40	27	159	6	87.0%	85.5%	60.0%	96.4%	86.3%	0.710
	**40**	36	7	179	10	78.3%	96.2%	84.1%	94.7%	87.3%	0.811
	**50**	31	4	182	15	67.4%	97.8%	88.9%	92.4%	82.6%	0.767
	**60**	28	0	186	18	60.9%	100%	100%	91.2%	80.5%	0.757
	**70**	24	0	186	22	52.2%	100%	100%	89.4%	76.1%	0.686
**External validation**	**30**	61	78	28	4	93.8%	26.4%	43.9%	87.5%	60.1%	0.598
	**40**	57	41	65	8	87.7%	61.3%	58.2%	89.0%	74.5%	0.699
	**50**	51	23	83	14	78.5%	78.3%	68.9%	85.6%	78.4%	0.734
	**60**	19	5	101	46	29.2%	95.3%	79.2%	68.7%	62.3%	0.427
	**70**	6	0	106	59	9.2%	100.0%	100.0%	64.2%	54.6%	0.169

TP: True Positive; FP: False Positive; TN: True Negative; FN: False Negative; PPV: Positive Predictive Value; NPV: Negative Predictive Value.

Supplementary Table S5. Area Under the Curve (AUC) performance of the derived score in the derivation and external validation cohorts, stratified by quartile of year of enrollment

**Year of enrollment**	**Q1**	**Q2**	**Q3**	**Q4**
**Derivation**	0.90 (0.80-0.98)	0.91 (0.79-0.98)	0.92 (0.69-0.98)	0.92 (0.78-0.99)
**External validation**	0.80 (0.56-0.93)	0.79 (0.70-0.92)	0.81 (0.61-0.85)	0.82 (0.66-0.90)

All values are estimate (95% confidence interval)
All p-value from Venkatraman's test for two unpaired ROC curves were non-significant.

Supplementary Table S6. Definitions

Variable	Definition
Heart failure	Defined according to the European Society of Cardiology (ESC) HF guidelines.
Arterial hypertension	Defined according to the European Society of Hypertension guidelines.
Obesity	Body mass index (BMI) ≥ 30 kg/m$^{2.}$
Diabetes	History of diabetes, use of any anti-diabetic medication, or the presence of fasting blood glucose ≥126 mg/dL confirmed on two different occasions.
Chronic Kidney disease	Defined as an estimated glomerular filtration rate (eGFR) <30 mL/min/1.73 m^2 (stages 4-5) according to the CKD Epidemiology Collaboration (CKD-EPI).
Ischemic heart disease	Defined as a history of myocardial infarction, percutaneous coronary revascularization, or coronary artery bypass graft.
Active infection	Defined as either the laboratory detection of the pathogen or its antigens, or the presence of clinical symptoms and elevated inflammatory markers.
Systemic disease	Defined as a rheumatologic condition affecting multiple organs, tissues, or systems throughout the body (i.e. lupus and rheumatoid arthritis).
Echocardiography	Echocardiography was performed and analyzed according to international recommendations. In brief, the following echocardiographic parameters were obtained: left ventricular end-diastolic diameter, left ventricular end-diastolic and end-systolic volumes, ejection fraction, diastolic function, and the presence of apical sparing at the global longitudinal strain evaluation. Echocardiographic examinations were digitally recorded and blind-read offline by expert readers using dedicated workstations.

Cardiac magnetic resonance	CMR was performed using a 1.5- scanner (Achieva; Philips Healthcare, Best, the Netherlands) equipped with a dedicated phased-array cardiac coil in accordance with current recommendations. The following CMR parameters were obtained: left and right ventricular end-diastolic and end-systolic volumes, left and right ventricular ejection fraction, stroke volume, hypertrophy, and pericardial disease. CMR examinations were digitally recorded and blind-read offline by expert readers using dedicated workstations.
Hypertrophy	Hypertrophy was defined based on the following criteria: (a) wall thickness >15 mm (or >13 mm in familial hypertrophic cardiomyopathy) in the absence of other causes; (b) a septal-to-lateral wall thickness ratio >1.3; or (c) an apical-to-basal wall thickness ratio ≥1.3 to 1.5 in apical hypertrophic cardiomyopathy.
Late gadolinium enhancement	To assess the presence of late gadolinium-enhancing (LGE) lesions, inversion recovery sequences were obtained 10 to 15 minutes after intravenous injection of 0.2 mmol/kg gadolinium. The location of LGE lesions was determined based on the 16-segment model, with the left ventricle divided into anterior (segments 1, 7, 13), septal (segments 2, 3, 8, 9, 14), inferior (segments 4, 10, 15), and lateral (segments 5, 6, 11, 12, 16) segments (**Supplementary Figure S1**). The left and right atria were collectively referred to as “atria” and were considered positive for LGE lesions if at least one of the two demonstrated LGE lesions.

Supplementary Figure S1. ROC curves for the derived score in the training set using 10-fold cross-validation and in the testing set.

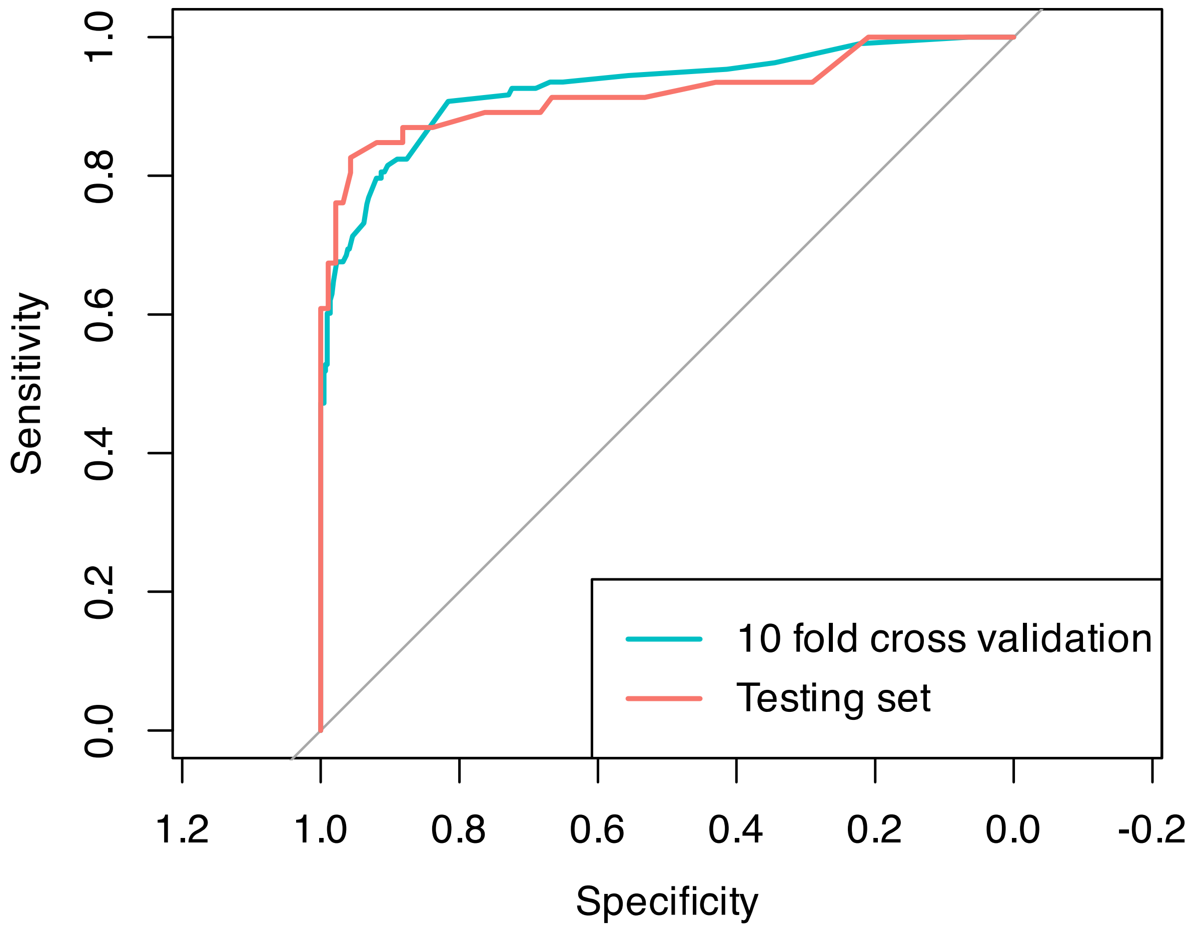

Supplementary Figure S2. Decision-Curve Analyses in the external validation cohort for the overall diagnosis (on the left) and non-amyloidosis-positive endomyocardial biopsies (on the right)

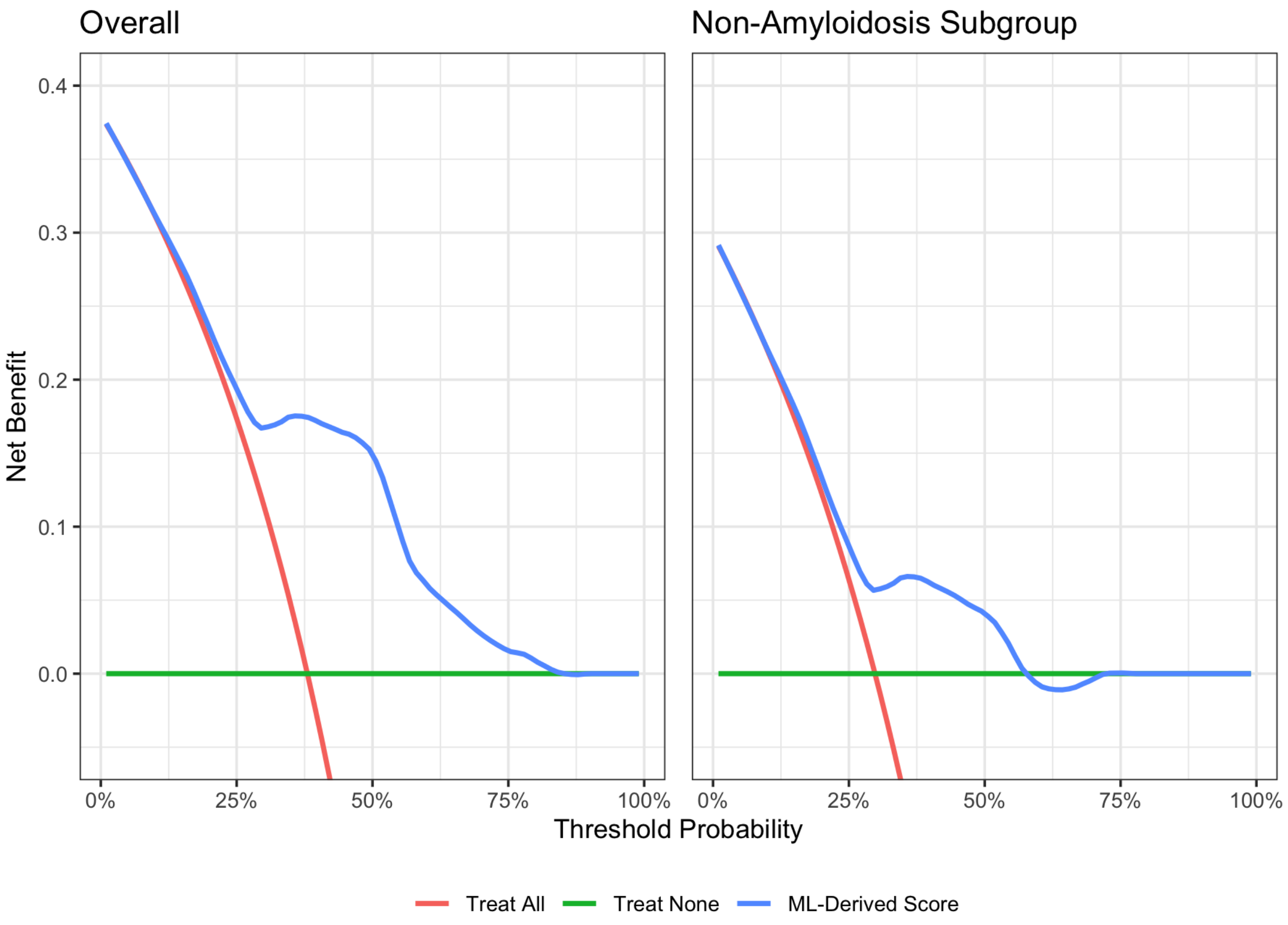

Supplementary Figure S3. Comparison between the full random forest model and the point score in the internal validation cohort.

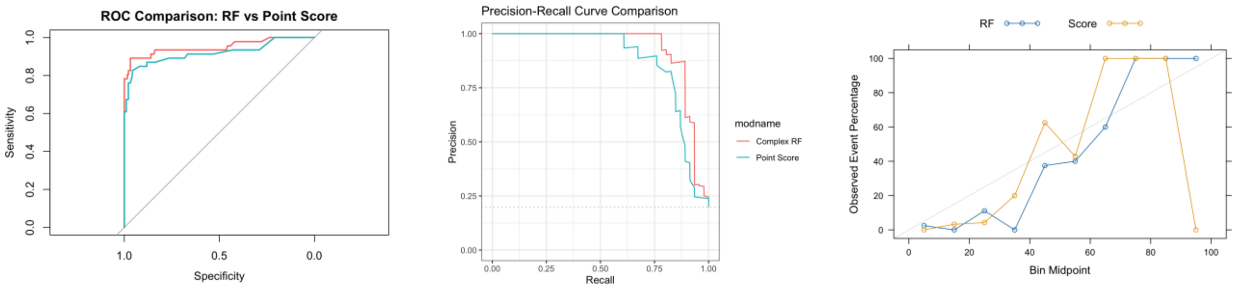

Supplementary Figure S4. Comparison between the full random forest model and the point score in the external validation cohort.

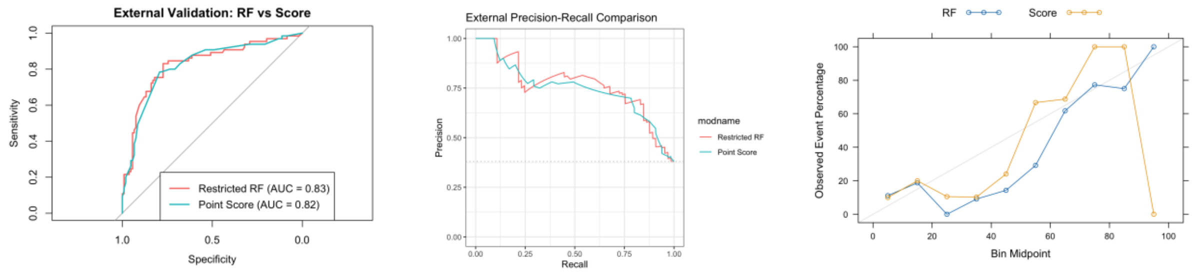

Supplementary Figure S5. Missingness over Time

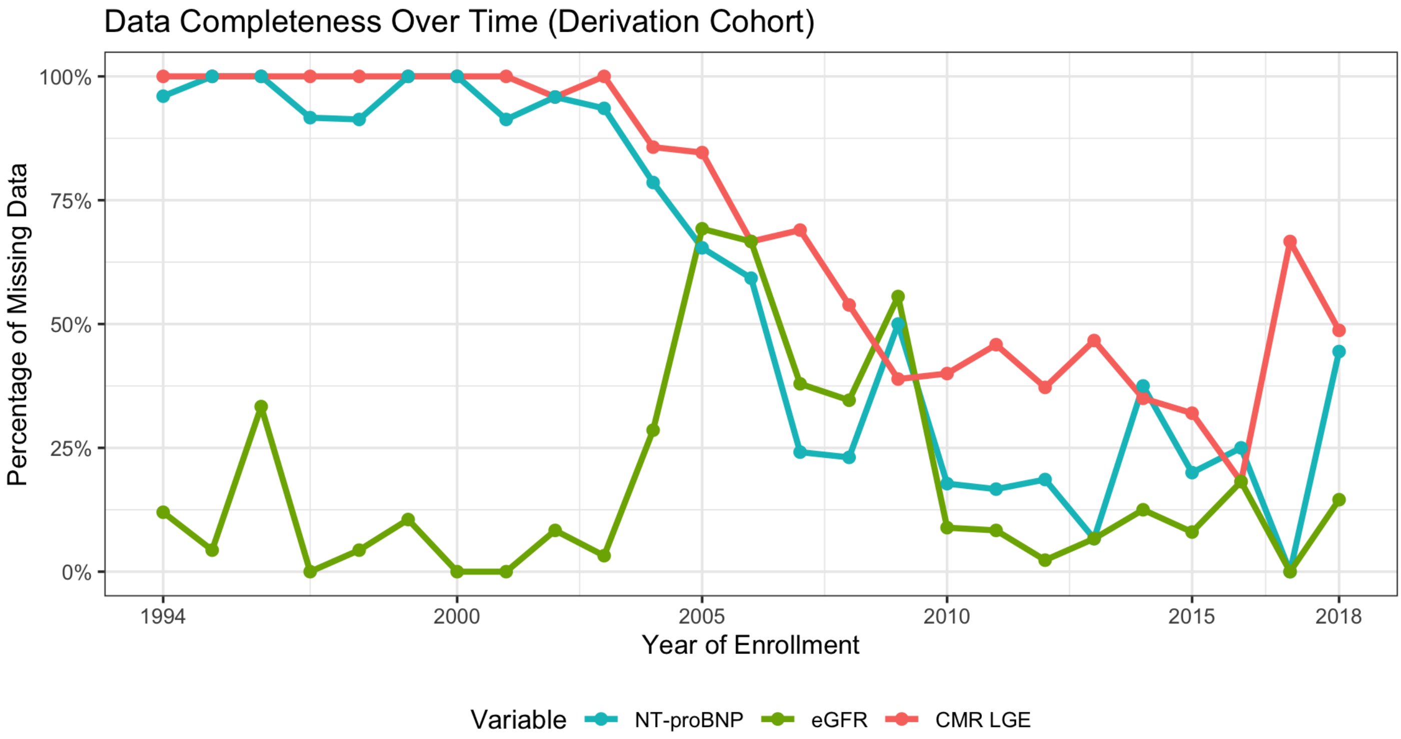

Supplementary Figure S6. 16-segment model used for cardiovascular magnetic resonance analysis

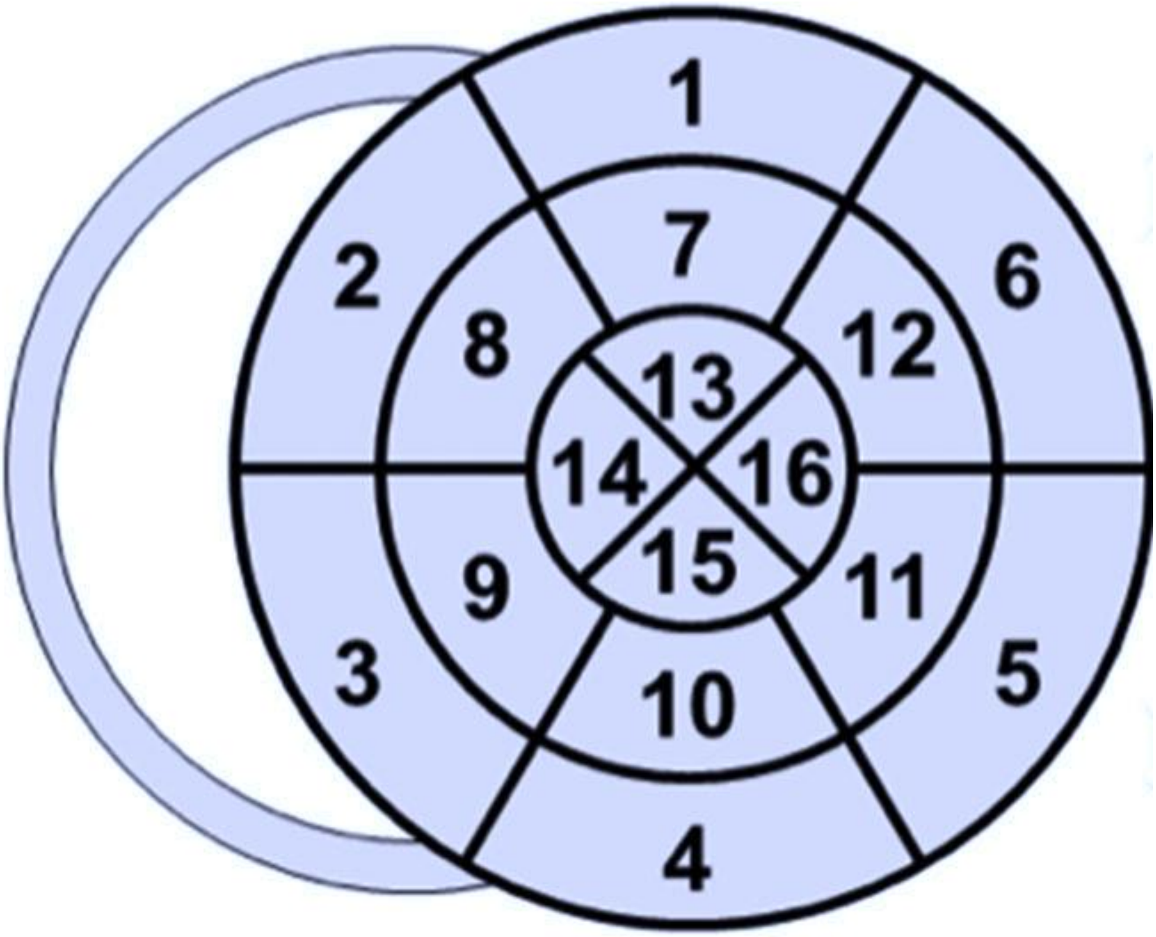

Segments:
1 – basal anterior
2 – basal anteroseptal
3 – basal inferoseptal
4 – basal inferior
5 – basal inferolateral
6 – basal anterolateral
7 – mid anterior
8 – mid anteroseptal
9 – mid inferoseptal
10 – mid inferior
11 – mid inferolateral
12 – mid anterolateral
13 – apical anterior
14 – apical septal
15 – apical inferior
16 – apical lateral
